# Stabilization of Homoserine-O-Succinyltransferase (MetA) Decreases the Frequency of Persisters in *Escherichia coli* under Stressful Conditions

**DOI:** 10.1371/journal.pone.0110504

**Published:** 2014-10-17

**Authors:** Elena A. Mordukhova, Jae-Gu Pan

**Affiliations:** Superbacteria Research Center, Korea Research Institute of Bioscience and Biotechnology (KRIBB), Daejeon, Korea; University of Florida, United States of America

## Abstract

Bacterial persisters are a small subpopulation of cells that exhibit multi-drug tolerance without genetic changes. Generally, persistence is associated with a dormant state in which the microbial cells are metabolically inactive. The bacterial response to unfavorable environmental conditions (heat, oxidative, acidic stress) induces the accumulation of aggregated proteins and enhances formation of persister cells in *Escherichia coli* cultures. We have found that methionine supplementation reduced the frequency of persisters at mild (37°C) and elevated (42°C) temperatures, as well as in the presence of acetate. Homoserine-*o*-succinyltransferase (MetA), the first enzyme in the methionine biosynthetic pathway, is prone to aggregation under many stress conditions, resulting in a methionine limitation in *E. coli* growth. Overexpression of MetA induced the greatest number of persisters at 42°C, which is correlated to an increased level of aggregated MetA. Substitution of the native *metA* gene on the *E. coli* K-12 WE chromosome by a mutant gene encoding the stabilized MetA led to reduction in persisters at the elevated temperature and in the presence of acetate, as well as lower aggregation of the mutated MetA. Decreased persister formation at 42°C was confirmed also in *E. coli* K-12 W3110 and a fast-growing WErph+ mutant harboring the stabilized MetA. Thus, this is the first study to demonstrate manipulation of persister frequency under stressful conditions by stabilization of a single aggregation-prone protein, MetA.

## Introduction

A small subpopulation of bacterial cells, designated persisters, which are able to survive lethal antibiotic treatment and produce a new population of antibiotic-sensitive cells genetically identical to the originals was first described by Joseph W. Bigger [1]. Persistence as a phenomenon of multi-drug tolerance without genetic changes has been found in various bacterial species: *Escherichia coli*, *Bacillus anthracis*, *Pseudomonas aeruginosa*, *Staphylococcus aureus, Gardnerella vaginalis*, *Salmonella enterica*, *Acinetobacter baumannii, Bordetella petrii* and *Mycobacterium tuberculosis* [2], [3], [4], [5], [6], [7], [8]. Because of the potentially harmful role of these bacteria in acute and chronic infections, an understanding of the nature of persistence is important to increase the efficiency of antibiotic treatment.

Persistence arises from the dormant state when the bacterial cells are metabolically inactive [3]; the level of translation is greatly reduced [9], resulting in arrested protein biosynthesis [10]. The frequency of persisters varies depending on the growth phase (from 0.0001–0.001% in exponential-phase to 1% in stationary-phase cultures), the age of the inoculum and the medium [11], [12], [13]; however, the “dormant” status of persisters was challenged by Orman and Brynildsen, who showed that dividing cells also gave rise to persisters, though to a lesser extent than non-dividing cells, and concluded that persistence was far more complex than dormancy [14]. The bacterial stress response to unfavorable environmental factors (nutrient, oxidative, heat and envelope stresses) also promotes reduced antibiotic susceptibility [15]. For example, the survival of heat-stressed *Acinetobacter baumannii* and *P. aeruginosa* increased in the presence of aminoglycosides or β-lactams [16], [17]. *E. coli* cells exposed to thermal stress accumulated a large number of aggregated proteins [18]. Leszczynska *et al*. showed that an increased level of protein aggregates in *E. coli* stationary-phase cells was strongly correlated with a higher frequency of persister formation [19]. In this context, we asked whether the inherently unstable MetA affects the formation of *E. coli* persisters under normal or stressful conditions. Homoserine *o*-succinyltransferase (MetA), the first enzyme in the methionine biosynthetic pathway [20], starts unfolding at 25°C *in vitro* and completely aggregates at temperatures of 44°C and higher, resulting in methionine limitation of *E. coli* growth [21]. MetA was found to be extremely sensitive to many stress conditions (e.g., thermal, oxidative or weak-organic-acid stress) [22], [23].

In this study, we have shown that exogenous methionine reduced the frequency of persister cells in the strain *E. coli* K-12 WE at mild (37°C) or elevated (42°C) temperatures, as well as in the presence of sodium acetate. Overexpression of MetA resulted in increased persister formation at 42°C and an enhanced level of aggregated MetA. Stabilized MetA mutant accelerated growth in the WE strain at the higher temperature (44°C) and in the presence of sodium acetate, decreased the frequency of persisters under heat and weak-acidic conditions and was less aggregation-prone. Strain W3110 and fast-growing mutants of strain WE expressing the wild-type and stabilized MetAs yielded similar results.

We showed the influence of a single aggregation-prone protein on persister formation in *E. coli* K-12 cells. Generally, our experiments confirmed that the stress response and dormancy appeared to be alternative strategies for cell survival [24].

## Materials and Methods

### Bacterial strains, media and culture conditions

The strains and plasmids employed in this study are listed in Table 1. *E. coli* strains were grown in minimal M9 medium [25] supplemented with glucose (0.2%) or in rich LB medium (Difco, San Jose, USA). Antibiotics were used at the following concentrations: ampicillin, 100 µg/ml, ofloxacin, 5 µg/ml, and kanamycin, 25 µg/ml. L-methionine was added to the medium to a final concentration of 50 µg/ml. Growth of *E. coli* strains in M9 glucose medium at different temperatures was studied in a TVS126MB automatic growth-measuring incubator (Advantec MFS Inc., Tokyo, Japan). The specific growth rate (µ, h^−1^) was calculated through linear regression analysis of ln(X/X_0_) data using Sigma Plot software, where the initial OD_600_ (X_0_) was 0.15 at the zero time point and X represents the OD_600_ values measured every 10 min in an exponentially growing culture for 1 h.

**Table 1:** Strains and plasmids used in this study.

Strain or plasmid	Relevant description	Source or reference
***Escherichia coli***		
DH5α	*F-,supE44 hsdR17 recA1 gyrA96 endA1 thi-1 relA1 deoR λ-*	[25]
W3110	F-, *λ* ^−^, *IN(rrnD-rrnE)1*, *rph-1*	KCTC
ATCC 9637 (W)	Wild -type	ATCC
JW3973	F-, Δ*(araD-araB)567*, Δ*lacZ4787(::rrnB-3)*, *λ* ^−^,rph-1, Δ*metA780::*kan, Δ(*rhaD-rhaB)568, hsdR514*	Keio collection National Institute of Genetics, Japan
JW0195	F-, Δ*(araD-araB)567*, Δ*lacZ4787(::rrnB-3)*, *λ* ^−^,rph-1, Δ*metN724::*kan, Δ(*rhaD-rhaB)568, hsdR514*	Keio collection National Institute of Genetics, Japan
WE	JW3973 carrying the wild-type *metA* gene	[27]
WE-LYD	WE carrying the *metA* gene with I124L, I229Y and N267D substitutions	[29]
W3110-LYD	W3110 carrying the *metA* gene with I124L, I229Y and N267D substitutions	This study
WE-_ P_BADMetA	WE carrying the wild-type *metA* gene under _P_BAD promoter, *kan*	This study
WErph^+^	WE carrying the *rph* gene from the strain *E. coli* ATCC 9637	This study
WE-LYDrph^+^	WE-LYD carrying the *rph* gene from the strain *E. coli* ATCC 9637	This study
BL21(DE3)	*F- ompT hsdS_B_(r-_B_m-_B_) gal dcm(DE3)*	Novagen (Billerica, USA)
**Plasmids**		
pCP20	ts rep,*[cI857] (λ;* ts), Ap^r^, *cat*, *[FLP]*	[26]
pKD13	oriR6γ, tL3LAM(Ter), Ap^r^, rgnB(Ter), *kan*	[26]
pKD46	*λ* Red (*gam bet exo) ara C rep101*(Ts), Ap^r^	[26]
pUC18	Cloning vector, Ap^r^	Laboratory stock
pET22b/MetA	Expression vector contains the wild-type *metA* gene, Ap^r^	[24]
pET22b/MetA-LYD	Expression vector contains the *metA* gene withI124L,I229Y and N267D substitutions, Ap^r^	This study
pBAD/HisA	Expression vector, Ap^r^	Invitrogen (Grand Island, USA

Ap^r^, ampicillin resistance; *kan*, kanamycin resistance gene.

### Construction of the fast-growing strains WErph^+^ and WE-LYDrph^+^

The native *rph-1* gene in the WE strain was replaced with the chloramphenicol-resistance gene using the λ Red recombination system [26]. A disruption cassette was synthesized through PCR with forward primer RG1 (*GGAAGTCCGTATAATGCGCAGCCACATTTGTTTCAAGCCGGAGATTTCAATATG* GTTGGCAGCATCACCCGAC), reverse primer RG2 (*GCGACTCATCAGTCGCCTTAAAAATCAGTTTGCCAGCGCCGCCTTCTGCGTCGC* GTAGCACCAGGCGTTTAAGG), Vent polymerase (NEB, Ipswich, USA) and the plasmid pACYC184 (NEB, Ipswich, USA) as a template (homologous sequences are shown in italics). The Δ*rph::cat* mutant of strain WE-LYD was obtained through transduction with P1*vir* using the WEΔ*rph* donor strain. For the *rph-kan* cassette construction, the kanamycin-resistance gene from the plasmid pKD13 [26] was cloned in the HindIII/AccI sites of pUC18 to generate the pUC18-Kan plasmid. The *rph* gene was amplified from *E. coli* ATCC 9637 genomic DNA using the primers RG3 (CGCCTCGGATCCGGAAGAAAAATGCCGCTCTG) and RG4 (GTTAAAGCAGTACGGCAGGTC) and cloned in the BamH1 site of pUC18-Kan resulted in the pUC18Rph-Kan plasmid which was used for the *rph-kan* cassette amplification with the primers RG5 (CGTTCATTGCCCACTCCATGTG) and RG6 (GAATCCACCAACGCTTCAGC). The 3.5-kb PCR product was transferred into WEΔ*rph*(pKD46) using the λ Red recombination system [26]. Strain WE-LYDrph^+^ was generated by *P1vir* phage transduction with the WErph^+^ donor strain. The kanamycin-resistance gene was eliminated from strains WErph^+^ and WE-LYDrph^+^ upon exposure to plasmid pCP20-encoded FLP recombinase [26]. The *rph* gene from the genomic DNA of strains WErph^+^ and WE-LYDrph^+^ was synthesized using the primers RG7 (GTCATACTGCGGATCATAGACG) and RG8 (GTTAAAGCAGTACGGCAGGTC), followed by sequencing with the primers RG9 (GGAGAGGTGGAAGGATTATAGC) and RG10 (GAATCCACCAACGCTTCAGC).

### Substitution of the native σ^32^ and σ^7^° promoters drove *metA* gene expression by the arabinose-inducible_ P_BAD promoter on the *E. coli* WE-strain chromosome

A two-step PCR procedure was used to construct the _P_BAD-*metA* cassette. The _P_BAD promoter was amplified from the template plasmid pBAD/HisA (Invitrogen, Grand Island, USA) using Vent polymerase (NEB, Ipswich, USA) with a first pair of primers, bad1 (CATACTCCCGCCATTCAGAGAAG) and bad2 (GTCCGGCACACGAATCGGCATGGTTAATTCCTCCTGTTAGC). The *metA* gene was synthesized using a second pair of primers, bad3 (GCTAACAGGAGGAATTAACCATGCCGATTCGTGTGCCGGAC) and bad4 (CGCCTCAGATCTCGTATGGCGTGATCTGGTAGAC), with *E. coli* WE genomic DNA as a template. The PCR products from the two first reactions were then used as templates in a second PCR with the primers bad1 and bad4. The resulting PCR product was digested with BglII and cloned into HincII-BamHI sites of the plasmid pUC18-Kan. The *kan*-_P_BAD-*metA* cassette was synthesized through PCR with the template plasmid pUC18-Kan-_P_BAD-*metA,* Vent polymerase (NEB, Ipswich, USA) and a pair of primers, bad5 (GAATACTAATAACCATTTTCTCTCCTTTTAGTCATTCTTATATTCTAACG CTTGAGCGATTGTGTAGGCTG) and bad6 (CGTATGGCGTGATCTGGTAGACGTAATAGTTGAGCCAG), then gel-purified and transferred into freshly prepared *E. coli* WE (pKD46) cells via electroporation, as described previously [26]. The *kan*-_P_BAD-*metA* cassette was synthesized from the genomic DNA of kanamycin-resistant clones and sequenced.

### Purification of MetA and differential scanning calorimetry

The MetAs were purified as described previously [27] in the presence of an EDTA-free Halt protease-inhibitor cocktail (Pierce, Rockford, USA). The thermal stabilities of the MetAs were measured calorimetrically over a temperature interval of 15–90°C at a scan rate of 90°C/h with a VP-DSC calorimeter (MicroCal, LLC, Northampton, USA) using 50 µM of protein in a 50 mM K-phosphate buffer (pH 7.5). Three scans were obtained using independent protein preparations.

### Purification of soluble and insoluble protein fractions

Cultures were grown in 50 ml of M9 glucose medium for 16 h or in LB medium for 24 h at 37°C or 42°C. Soluble and insoluble protein fractions were purified as previously described [21], [28] in the presence of EDTA-free Halt protease-inhibitor cocktail (Pierce, Rockford, USA). Three micrograms of total protein from the soluble fraction and 10 µl of the insoluble fraction were subjected to 12% SDS-PAGE, followed by Western blotting using rabbit anti-MetA antibody [29]. The MetA in the samples was quantified through densitometry using WCIF ImageJ software.

### Persister detection assay

Bacteria grown overnight in M9 glucose medium for 16 h or in LB medium for 24 h were diluted to an OD_600_ of 0.1 in fresh medium (M9 glucose or LB) supplemented with ampicillin (200 µg/ml) or ofloxacin (5 µg/ml) and incubated at 37°C for 10 hours. Samples were taken every hour and plated on LB agar for colony counting. The values represent the means of three independent experiments. The frequency of persister formation was determined as the relationship between the CFU of surviving bacteria and the total CFU before the addition of antibiotics. The error bars indicate the standard errors.

### Statistical analyses

The significance of differences between mean values of two measured parameters was assessed using two-tailed t test with unequal variances. Differences were considered significant when the *P* value was <0.05.

## Results

### Exogenous methionine decreased frequency of persisters in the *E. coli* cells at mild and elevated temperatures

**Figure 1:**
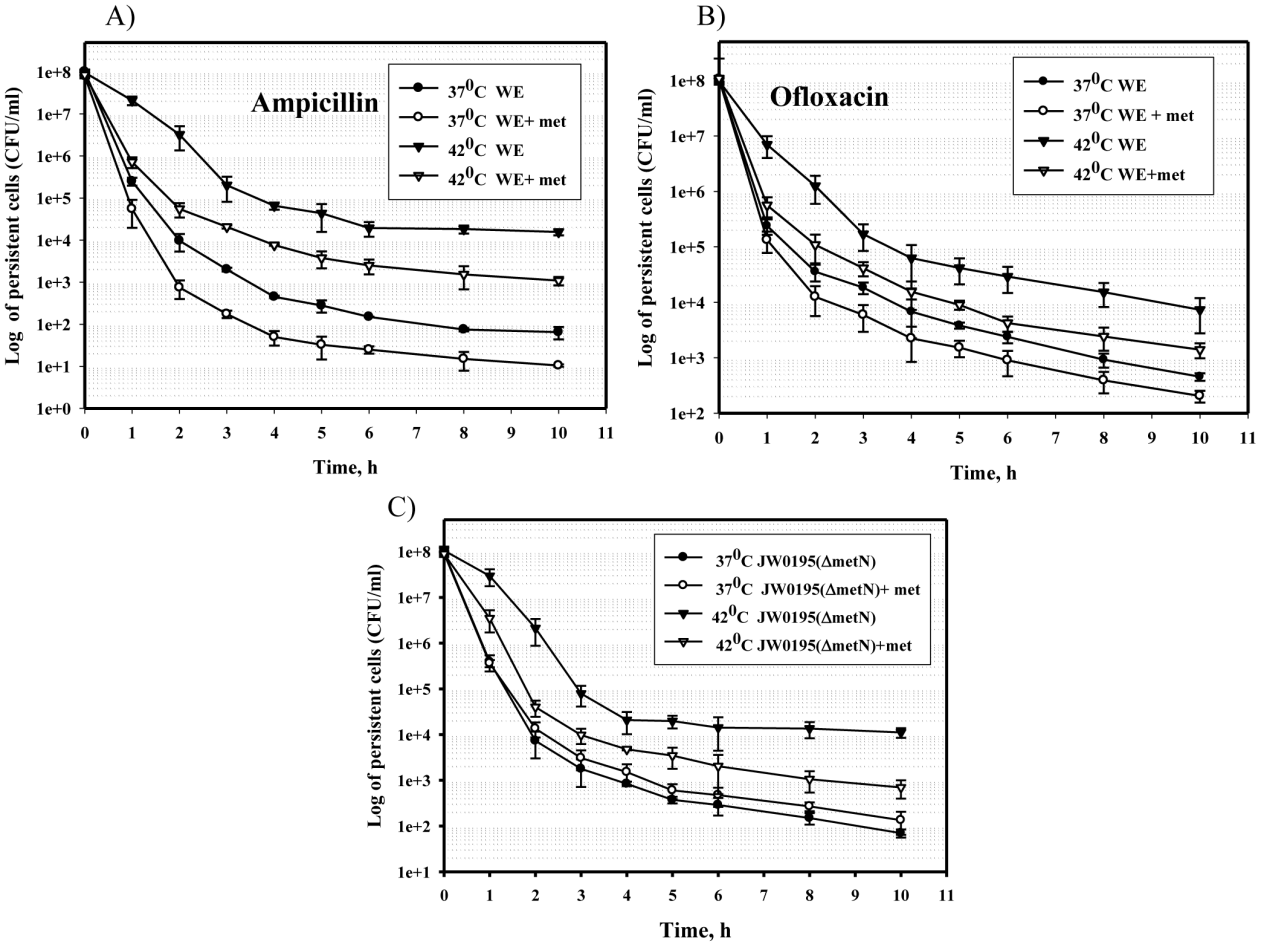
Effect of L-methionine on the frequency of persisters at different temperatures. The 16-h cultures of the strains WE (A, B) and JW0195 (C) grown in M9 glucose medium with or without L-methionine (50 µg/ml) at 37 or 42°C were diluted to an OD_600_ of 0.1 in fresh M9 glucose medium supplemented with ampicillin (A, C) or ofloxacin (B) and incubated at 37°C for 10 hours. Samples were analyzed as described in the Materials and Methods.

Previous findings have revealed that *E. coli* growth in the defined medium was impaired at elevated temperatures due to methionine limitation resulting from the extreme inherent instability of the first enzyme in the methionine biosynthetic pathway, MetA [20], [30]. Because the MetA was completely aggregated at 44°C [21], we studied the effect of temperature and methionine supplementation on persister formation in *E. coli* K-12 WE cells. Strain WE of *E. coli* K-12 grown in M9 glucose medium at 37 and 42°C for 16 h was treated with ampicillin, and the frequency of persisters was determined by plating samples on LA plates (Figure 1A). To distinguish persisters from resistant mutants, the colonies were replica plated on LA plates supplemented with ampicillin. No colonies grew in the presence of ampicillin.

As seen in Figure 1A, the time-kill curves of the cells grown at 37°C and at 42°C were typically biphasic, representing exponential death of the non-persistent cells, followed by a slower death rate for the persisters [31]. Because an increased frequency of persisters is linked to a slow-growing state [32], we compared the specific growth rates of the WE strain at mild vs. higher temperatures and did not detect slower growth at 42°C (Table S1). We hypothesized that more than 150-fold increase in the frequency of persisters at 42°C (p<0.05) resulted from an increased level of aggregated proteins. As homoserine *O*-succinyltransferase (MetA), which catalyzes the first unique step in the *de novo* methionine biosynthetic pathway, is inherently unstable and prone to aggregation [21]–[23], we determined the number of persisters in cultures in the presence of methionine (Figure 1A) when the genes involved in methionine biosynthesis were repressed [33]. Methionine supplementation reduced the frequency of persisters tolerant to ampicillin by 6–9 times at 37°C and by 9–15 times at 42°C (p<0.05) compared to methionine-free medium (Figure 1A). Because bacterial killing and persister formation with ampicillin as a beta-lactam antibiotic depend on the growth rate [32], [34], which would be affected by exogenous methionine [27], we examined the frequency of persisters tolerant to another antibiotic, ofloxacin, in cultures grown with or without methionine supplementation at 37°C and 42°C (Figure1B). Ofloxacin is a fluoroquinolone antibiotic that binds DNA gyrase and topoisomerase IV, leading to inhibition of bacterial cell division and cell growth [35]. Ofloxacin effectively kills bacteria regardless of the growth phase [36]. At elevated temperature (42°C), strain WE produced 16-fold more cells tolerant to ofloxacin than at 37°C (p<0.05) (Figure 1B). Methionine supplementation decreased the number of ofloxacin persisters 5–6 times at 37°C and 8 times at 42°C (p<0.05) (Figure 1B). Thus, exogenous methionine reduced the number of persisters at both higher and lower temperatures, regardless of the type of antibiotic used.

To confirm the effect of exogenous L-methionine on persister-cell formation, we obtained the time-kill curves of the mutant JW0195 (Δ*metN*) lacking the L-methionine ABC transporter MetN [37]. As seen in Figure 1C, provision of exogenous methionine to the JW0195(Δ*metN*) mutant did not affect the number of persister cells tolerant to ampicillin at 37°C. At 42°C, however, the number of persisters was 6–15 times lower in the presence of L-methionine than in methionine-free medium (p<0.05) (Figure 1C). We assume that at elevated temperature, *E. coli* cells defective in MetN biosynthesis may activate another L-methionine transport system, the genetically uncharacterized MetP system, [37], [38] to compensate for methionine deficiency, resulting in a lower persister level.

Thus, these results showed that the formation of persisters was dependent on the availability of methionine and might be linked to the solubility of MetA.

### The frequency of persister formation is correlated to the aggregation of the MetA

To determine whether the MetA participates in persistence, we compared the level of persistence to ampicillin in a pair of isogenic strains, JW9673(Δ*metA*) and WE harboring the wild-type *metA* gene [27], in LB medium at 37 and 42°C. The strains produced similar numbers of persister cells at each temperature (data not shown). One possible explanation is that the expression of methionine-biosynthetic genes was repressed by methionine [33], whose concentration in LB medium was estimated at approximately 6 mM [39], approximately 17 times higher than the amount used to supplement the M9 glucose medium. Secondly, deletion of the *metA* gene, like deletion of *rmf*, *relE*, or *mazF,* did not affect persister production [40]. Therefore, we examined the frequency of persistence when MetA was over-expressed.

**Figure 2:**
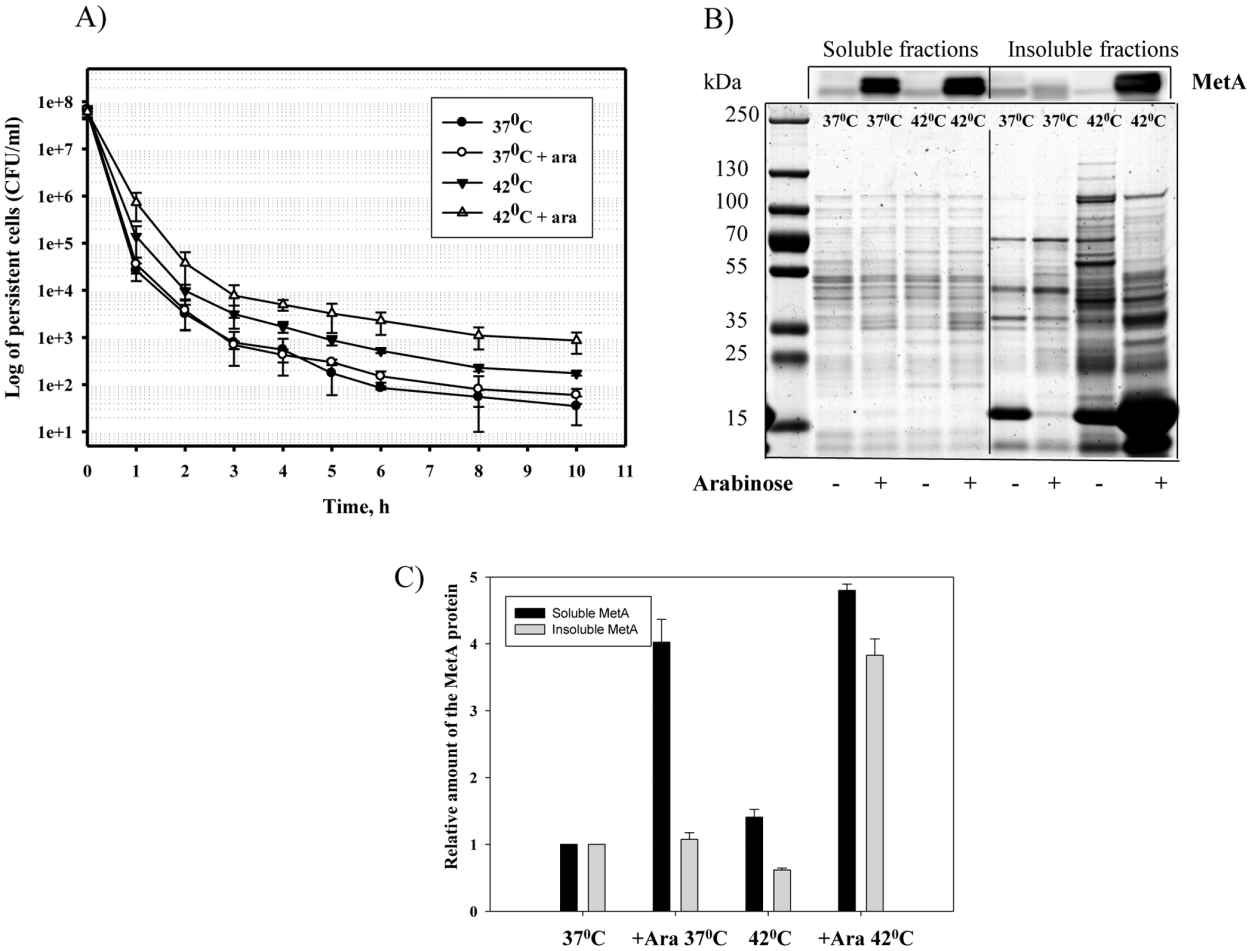
Effect of the MetA overexpression on persister formation at different temperatures. Strain WE harboring the *metA* gene under pBAD promoter was grown in LB medium at 37 or 42°C with or without arabinose (10 mM) for 24 h, diluted to an OD_600_ of 0.1 in fresh LB medium supplemented with ampicillin and incubated at 37°C for 10 hours. Samples were analyzed as described in the Materials and Methods (A). Soluble and insoluble protein fractions were isolated from the late-stationary phase cultures (24 h) grown in LB medium, subjected to 12% SDS-PAGE followed by Western blotting using rabbit anti-MetA antibody (B). The MetA in the samples was quantified through densitometry using WCIF ImageJ software. The MetA amount from the cells grown at 37°C without arabinose was set to 1 (C). The error bars represent the standard deviations of duplicate independent cultures.

Previous investigations have shown that *metA* gene expression increased up to 50 times during heat shock within 5 min of induction and increased 3–4 times in the presence of acetate [41], [42]. Expression of *metE* and *metC* remained unchanged during heat shock [41]. Evidence later showed that MetA had a strong tendency to unfold and aggregate at elevated temperatures [21], [22]. To test whether MetA over-expression and aggregation affect persister formation, the *metA* gene on the WE strain chromosome was placed under tight control of the arabinose- regulated pBAD promoter. The native *metA* promoters, σ^32^ and σ^7^° [43], were deleted from the chromosome. The frequency of persisters and MetA aggregation were studied in 24-h WE-pBADMetA culture grown in LB medium at 37 and 42°C with or without L-arabinose. At 37°C, we did not detect any difference in the numbers of persisters produced by induced and non-induced cultures (Figure 2A). At an elevated temperature (42°C), the WE-pBADMetA strain demonstrated 3-6-fold-higher persister frequency when the culture was non-induced (p<0.05), but arabinose induction increased the number of persisters approximately 10–25 times in comparison to culture at 37°C induced (p<0.05) (Figure 2A). Strain JW3973, which lacked the *metA* gene, was examined in terms of persister formation under the conditions described above. The frequency of persisters detected in the JW3973 strain was similar to that obtained in the non-induced culture of the WE-pBADMetA strain (data not shown).

Leszczynska *et al*. found that the number of persisters corresponded to the level of protein aggregation [19]. We detected increased aggregation in the cultures grown at 42°C compared to cells grown at 37°C (Figure 2B). This result may partially explain the higher persister frequency at the elevated temperature. We also examined the levels of soluble and insoluble MetA at 37 and 42°C in the non-induced and induced cultures (Figure 2B and 2C). At 37°C, the amount of soluble MetA was 4-fold higher in the arabinose-induced culture compared to the non-induced culture, whereas the relative amount of insoluble MetA was almost the same (Figure 2B and 2C). At 42°C, the soluble MetA content was 1.2 times that of both cultures at 37°C, but the aggregated MetA amount was 3.8 times higher in the presence of arabinose and 2 times lower without arabinose (Figure 2B and 2C). An insoluble protein that showed an intensive band around 15kDa in the SDS-PAGE gel (Figure 2B) was recognized with antibodies specific to MetA (data not shown). This protein is perhaps a product of MetA degradation that is carried out by ATP-dependent proteases [22]. Thus, these results showed the direct impact of MetA aggregation on persister-cell formation.

### A stabilized MetA mutant decreases the frequency of persisters at elevated temperatures

**Figure 3:**
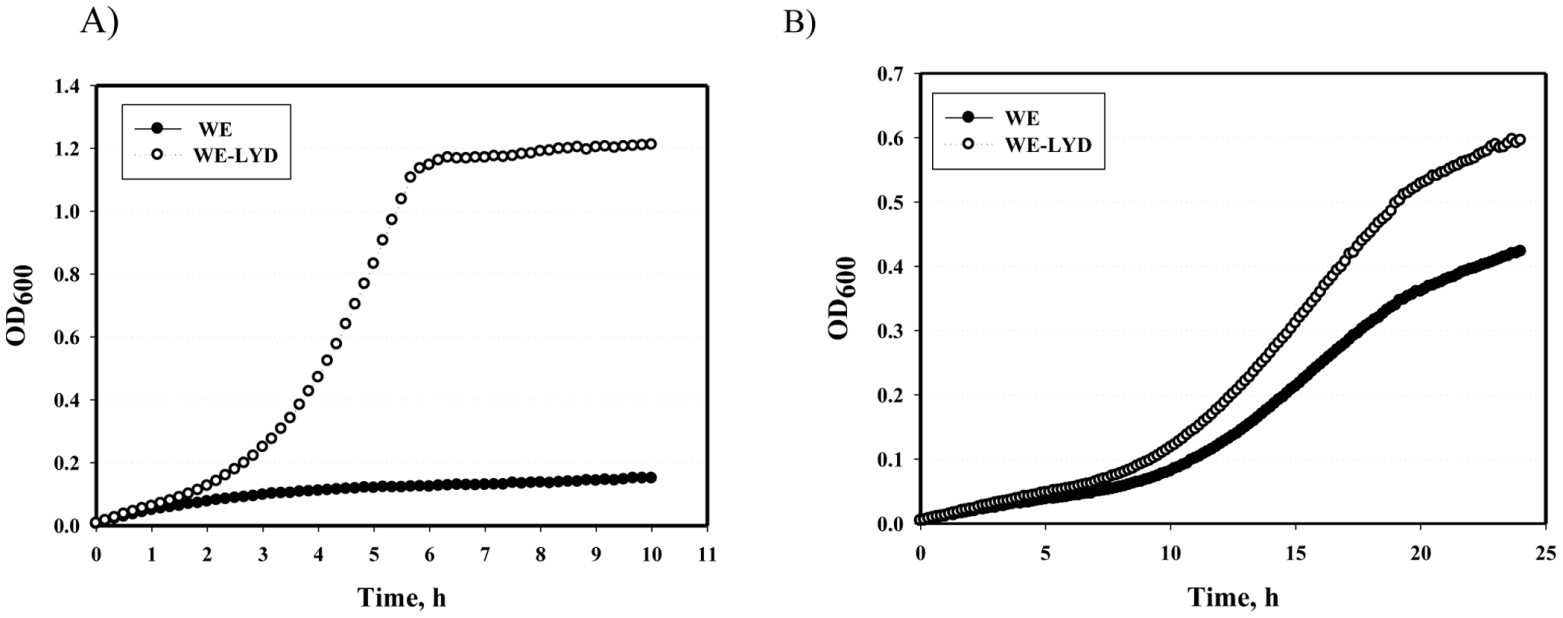
Influence of stabilized MetA protein on the *E.coli* WE strain growth under stressful conditions. The WE and WE-LYD strains were incubated in M9 glucose medium at 44°C for 10 h (A) or in M9 glucose medium (pH 6.0) supplemented with 20 mM sodium acetate at 37°C for 28 h (B) in an automatic growth-measuring incubator. The average of two independent experiments is presented.

As MetA aggregation increased persister production, we studied the effect of MetA stabilization on the persister frequency at mild (37°C) and elevated temperatures (42°C). Strain WE-LYD, which harbors three stabilizing mutations in MetA (I124L, I229Y and N267D), had been constructed previously [29] and showed accelerated growth at an elevated temperature (44°C) or in the presence of sodium acetate (Figure 3, Table S1). We measured the melting temperatures (*T*_m_) of the wild-type and mutant proteins using differential scanning calorimetry (DSC). Both of these proteins contain a C-terminal six-histidine tag and were purified as described in the Materials and Methods. Mutated MetA-LYD had a higher *T*_m_ than wild-type MetA (52.65±0.06°C and 47.07±0.01°C, respectively), evidence of the increased thermal stability of the MetA-LYD mutant.

**Figure 4:**
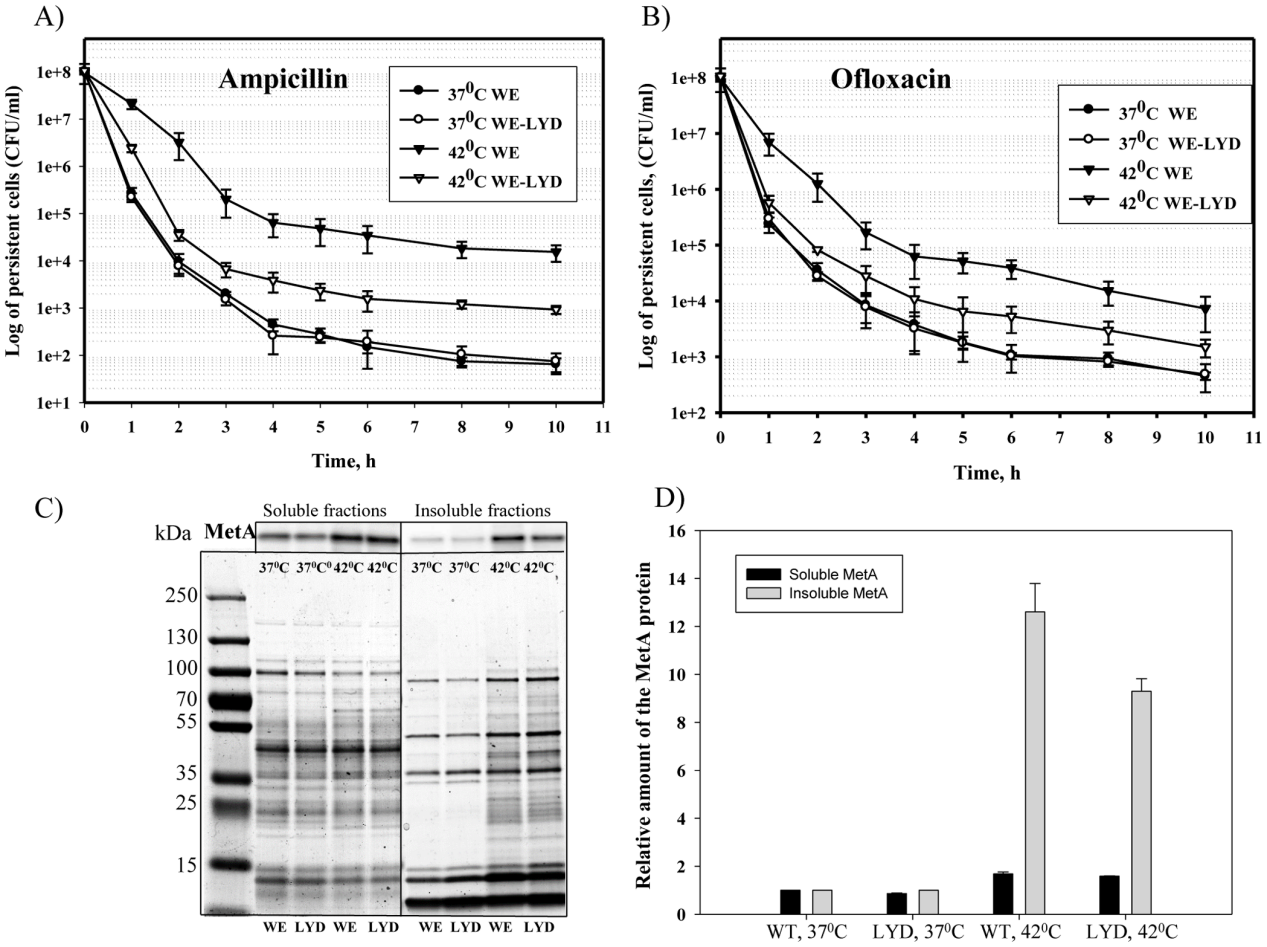
Dependence of persister formation on stabilized MetA protein. Overnight cultures of the strains WE and WE-LYD grown for 16 h in M9 glucose medium at 37 or 42°C were diluted to an OD_600_ of 0.1 in fresh M9 glucose medium supplemented with ampicillin (A) or ofloxacin (B) and incubated at 37°C for 10 hours. Samples were analyzed as described in the Materials and Methods. Soluble and insoluble protein fractions were purified from the cultures grown in M9 glucose medium at 37 or 42°C to an OD_600_ = 1.0, subjected to 12% SDS-PAGE followed by Western blotting using rabbit anti-MetA antibody (C). The MetA in the samples was quantified through densitometry using WCIF ImageJ software. The MetA amount from the WE cells grown at 37°C was set to 1 (D). The data are presented as the average of two independent experiments.

A pair of isogenic strains, WE and WE-LYD, was used for the study of persister formation at 37°C and 42°C in M9 glucose medium. Both strains displayed similar numbers of persisters at 37°C (Figure 4A). At 42°C, the frequency of increased in both strains (Figure 4A); however, the WE-LYD mutant strain formed 15–22 times fewer persisters than the WE harboring the wild-type MetA (p<0.05) (Figure 4A). A similar tendency was observed in the formation of persisters tolerant to ofloxacin; however, the difference in the number of persisters between the two strains decreased up to 5–8 times at 42°C (p<0.05) (Figure 4B).

We have detected 12.5-fold more aggregated wild-type MetA at 42°C compared to 37°C, but the level of stabilized MetA increased only 9.5 times (Figure 4C and 4D). The relative amount of soluble MetA was 1.5 times higher at 42°C (Figure 4C and 4D), consistent with previous findings that showed activation of *metA* transcription at elevated temperatures [41]. Strains WE and WE-LYD did not exhibit any difference in their specific growth rates at 37 and 42°C (Table S1), linking the finding that the highest level of persisters was formed by the WE strain at 42°C to an increase in the aggregate level of wild-type MetA.

### Lower persister frequencies correlate with MetA stabilization independently of strain or growth rate

To test whether the MetA stabilization influences the frequency of persisters in other *E. coli* strains, we substituted the native *metA* gene on the W3110 chromosome with the *metA*-LYD mutant and constructed fast-growing mutants of the WE and WE-LYD strains. Previous studies have shown that the genomes of *E. coli* K-12 strains MG1655 and W3110 harbor a GC deletion within the 3′-terminal part of the *rph* gene that causes partial auxotrophy for pyrimidines, resulting in a growth defect for K-12 strains [44]. As the *rph* gene from *E. coli* strain ATCC 9637 (W) does not contain this mutation, we substituted the defective *rph* gene in the K-12 WE and WE-LYD strains with the *rph* gene from the ATCC 9637 strain. The resulting WErph+ and WE-LYDrph+ mutant strains grew 13–15% faster at 37° and at 42°C than the parental strains (Table S1).

**Figure 5:**
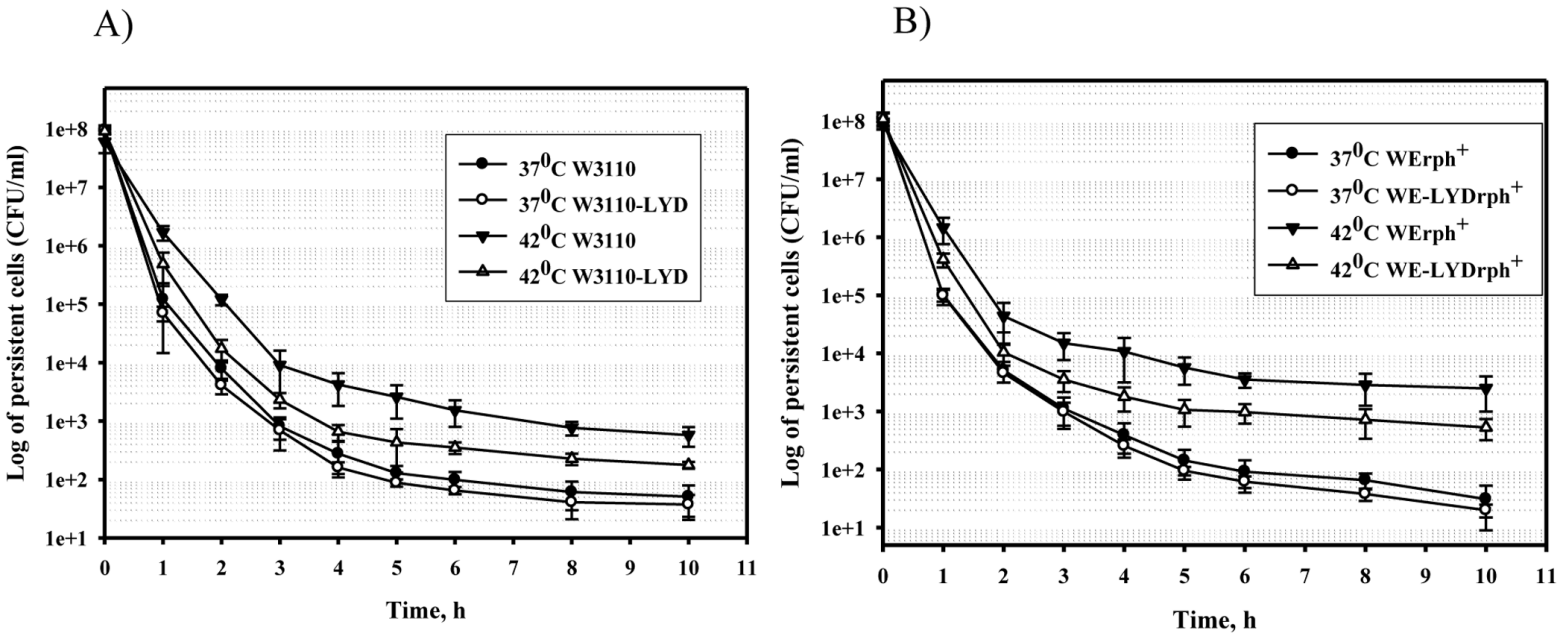
Stabilized MetA decreases the frequencies of persisters in different *E. coli* strains at elevated temperature. Cells of the strains W3110 and W3110-LYD (A), WErph^+^ and WErph^+^-LYD (B), grown overnight for 16 h in M9 glucose medium at 37 or 42°C, were diluted to an OD_600_ of 0.1 in fresh M9 glucose medium supplemented with ampicillin and incubated at 37°C for 10 hours. Samples were analyzed as described in the Materials and Methods.

Persister formation by two other pairs of isogenic strains, W3110/W3110-LYD, and WErph+/WE-LYDrph+ grown in M9 glucose medium at 37°C and 42°C followed the same tendency demonstrated earlier: no difference was detected at 37°C and more persisters were produced by the strain harboring wild-type MetA at 42°C (Figure 5A and 5B). Thus, these results confirmed our hypothesis that stabilization of highly unstable MetA reduces the frequency of persisters in *E. coli* strains at an elevated temperature.

### Stabilized MetA reduces persister formation in the presence of acetate

**Figure 6:**
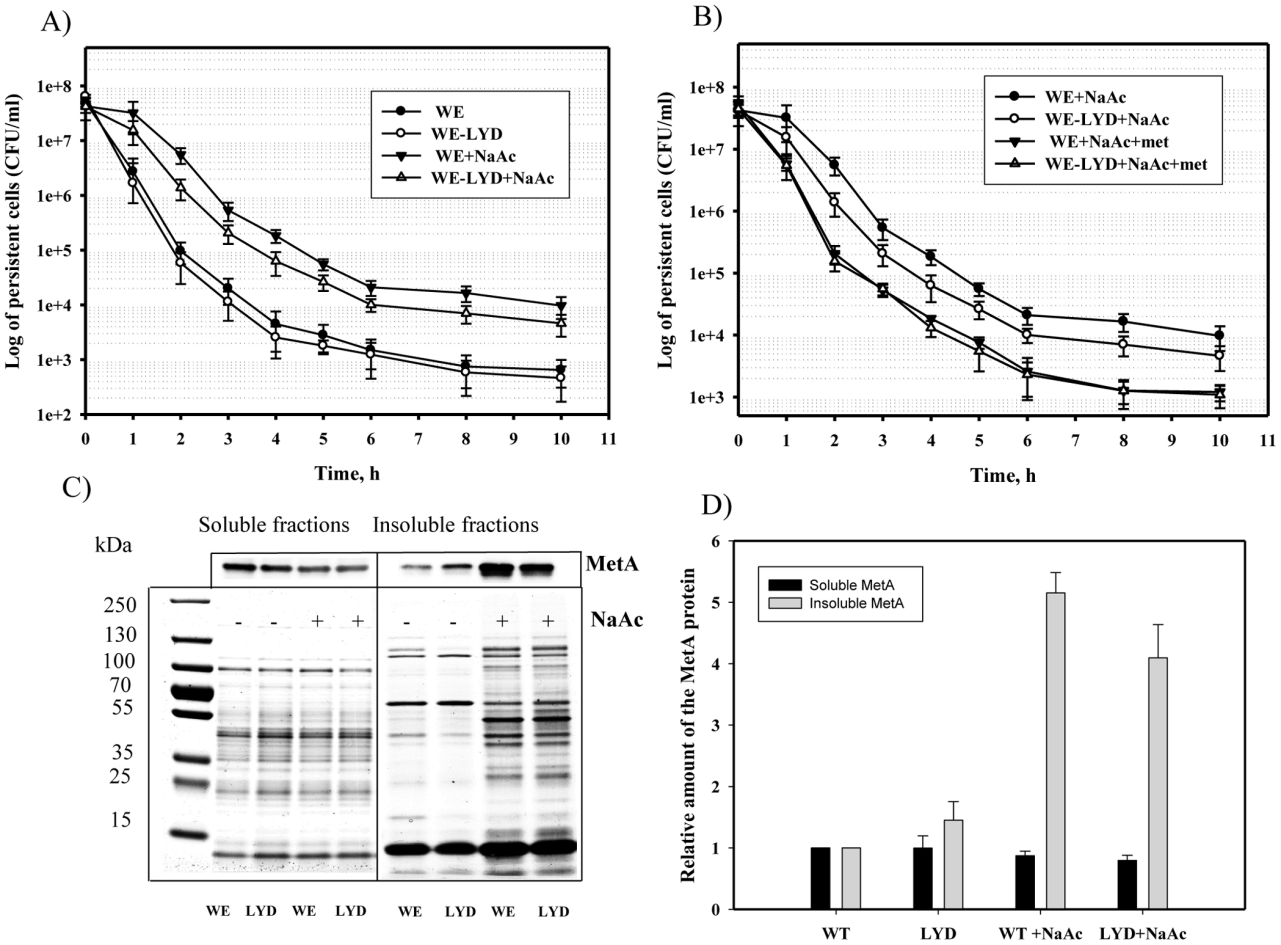
Effect of the stabilized MetA on the persister cell frequency under acidic conditions. Cultures of WE and WE-LYD grown for 16 h in M9 glucose medium (pH 6.0) at 37°C with or without sodium acetate (20 mM; A); with or without L-methionine (50 µg/ml) and in the presence of sodium acetate (20 mM; B) were diluted in fresh M9 glucose medium to an OD_600_ of 0.1, supplemented with ampicillin and incubated at 37°C for 10 hours. Samples were analyzed as described in the Materials and Methods. Soluble and insoluble protein fractions were purified from the 16 h-cultures grown in M9 glucose medium (pH 6.0) with or without sodium acetate (20 mM), and subjected to 12% SDS-PAGE, followed by Western blotting using rabbit anti-MetA antibody (C). The MetA in the samples was quantified through densitometry using WCIF ImageJ software. The amount of MetA in the WE cells grown without sodium acetate was set to 1 (D). The data are presented as the average of two independent experiments.

A previous study reported that acetate induced protein aggregation and increased the frequency of persisters in *E. coli* MC4100 culture [19]. Acetate treatment was found to alter significantly the expression of 86 genes including the *metA* gene whose expression was increased 3–4 times [42]. Supplementation of the medium with methionine partially relived the growth inhibition of E.coli caused by acetate [45], [46]. Because the stabilized-MetA mutant facilitated growth of the WE-LYD strain in the presence of acetate (Figure 3B), we tested persister formation by WE and WE-LYD cultures grown overnight in the M9 glucose medium (pH 6.0) supplemented with sodium acetate (20 mM) at 37°C. As seen in Figure 6A, acetate enhanced the frequency of persisters in both WE and WE-LYD cultures compared to acetate-free medium; however, the WE-LYD strain in the presence of acetate formed almost 2–4 times fewer persisters than the WE strain (p<0.05) (Figure 6A). Supplementation of the acetate-enriched medium with exogenous methionine reduced the frequency of persisters to a similar level in both tested strains compared to the methionine-free medium (Figure 6B). Increased numbers of persisters in acetate-enriched medium were accompanied by a higher aggregate level (Figure 6C). The amount of aggregated wild-type MetA increased 5-fold with acetate supplementation, and the amount of the MetA-LYD mutant increased 4-fold (Figure 6C and 6D). Thus, methionine enhances the susceptibility of *E. coli* to antibiotics, and an increase in stability of one protein (MetA) affects persister formation under weak-acidic conditions.

## Discussion

Interest in microbial persistence is rising; however, the mechanisms underlying persister formation are not fully understood. Populations containing higher numbers of dormant or slow-growing cells were highly persistent under antibiotic treatment, a phenomenon associated with decreased biosynthetic activity [9], [10]. Bacteria also recruit resistance determinants and induce antimicrobial-resistance mechanisms in response to diverse environmental stresses [15]. The genes involved in the heat and cold stress responses (*cspH*, *htrA*, *ibpAB*, *htpX*, and *clpB*) were upregulated in the cell samples with the higher frequencies of persisters [40]. These genes are overexpressed under stress conditions, but their role in antibiotic tolerance still has not been clearly explained [40]. Lon protease (annotated as the ATP-dependent heat shock protein) enhances the number of persisters when overexpressed [47]. In turn, cells lacking Lon protease, as well as the chaperones DnaK and DnaJ, reduced the formation of persistent cells [47], [48]. Overproduction of DnaJ stimulated the persistence of *E. coli* cells [49]. Increased expression of the heat-shock proteins DnaK, DnaJ and Lon was found at elevated temperatures and under other stressful conditions [50], [51], [52]. The DnaK system, consisting of the chaperones DnaK, DnaJ and GrpE together with ClpB, maintains proper protein folding, and the proteases Lon, Clp and HtrA degrade the protein aggregates that form at higher temperatures [18], [53]. Therefore, enhanced protein misfolding and aggregation resulted in overexpression of the heat-shock proteins, which may be linked to a higher persister frequency.

MetA is a heat-shock protein [41] that is highly unstable at elevated temperatures [21]–[23]. The MetA started to unfold *in vitro* at temperatures of approximately 25°C, with the maximum level of unfolding at 44°C, resulting in complete aggregation with any subsequent rise in temperature [21]. Indirect evidence suggests that MetA requires folding assistance from the DnaK chaperone system at mild and elevated temperatures [29], [54]. Aggregated MetA is also a substrate for the ATP-dependent cytosolic proteases Lon, ClpPX/PA and HslVU [22]. Thus, an accumulation of misfolded and/or aggregated MetA associated with increased expression of the chaperones and proteases may increase the level of persisters.

Methionine added to the culture medium to repress transcription of the methionine-biosynthetic genes [33] reduced the number of persisters at mild and elevated temperatures (Figure 1A and B). This result might be explained by the absence of two aggregation-prone proteins, MetA and MetE, from the methionine-biosynthesis pathway [18]. Methionine also stimulates *E. coli* growth in defined medium [27], decreasing persistence [55]. The higher cultivation temperature in the absence of methionine significantly increased the frequency of persisters, independent of the medium (Figures 1A and 2A). A similar effect was observed when the cells were grown in the acetate–enriched defined medium at the mild temperature (37°C; Figure 6A). In each case, increased persistence was associated with a higher level of protein aggregation (Figures 2B, 4C and 6C), which is consistent with a previous report by Leszczynska *et al*. [19], who have shown a correlation between the level of protein aggregates and the frequency of persisters. The amount of MetA in the insoluble protein fractions under stressful conditions was also significantly higher compared to the amounts produced by normally growing cells (Figures 2B, 2C, 4C, 4D, 6C and 6D). We found that the stabilized-MetA mutant had notably reduced persister frequency at the elevated temperature independent of strain (Figures 4A, 4B, 5A and 5B), as also seen in the presence of acetate (Figure 6A). The level of mutated MetA in the insoluble protein fractions from heat- and acid-treated cells was lower than the level of wild-type protein (Figures 4C, 4D, 6C and 6D). We suggest two causes of the decreased frequency of persisters generated by the strain with stabilized MetA. First, the stabilized-MetA mutant requires less assistance from chaperones to refold the misfolded protein and less Lon protease to degrade the aggregates, resulting in lower expression of these enzymes and thus in a reduced number of persisters under stress. Second, refolding and/or proteolysis of the denatured/aggregated MetA might be facilitated by inorganic polyphosphate (PolyP), a product of the polyphosphate kinase [23]. Lower levels of mutated MetA aggregates compared to the wild-type protein might cause a decrease in PolyP production, followed by reduced persister formation [56].

Increased persister formation with inherently unstable MetA raises an intriguing question: ‘Does inherently instable MetA favor *E. coli* survival under antibiotic challenge?’ If unstable MetA offers a selective advantage, more stable MetA may not evolve.

In summary, we found that the first enzyme in the methionine biosynthetic pathway, MetA, affects the level of persisters in *E. coli* under stressful conditions. A higher frequency of persisters was correlated with an increased amount of aggregated MetA. Stabilization of the unstable MetA enzyme resulted in decreased aggregation and thus in reduced persister formation at elevated temperature and in the presence of acetate.

Thus, we have shown a possibility to correct persister formation by manipulating the thermostability of the single enzyme, MetA.

## Supporting Information

Table S1 **Effect of stabilized MetA protein on** ***E.coli*** **growth at different temperatures or in the presence of sodium acetate.** Strains were grown in M9 glucose medium (pH 7.0 or 6.0) with or without sodium acetate (20 mM) in an automatic growth-measuring incubator at indicated temperatures for 24 h. The specific growth rate μ (h^−1^) was calculated through linear regression analysis of ln(X/X_0_) data with Sigma Plot software, where the initial OD_600_ (X_0_) was 0.1–0.15 at the zero time point, and X represents the OD_600_ values measured every 10 min in an exponentially growing culture over 1 h.
(DOC)
