# Supplementary material for: Stabilization of Homoserine-O-Succinyltransferase (MetA) Decreases the Frequency of Persisters in Escherichia coli under Stressful Conditions
Source: PLoS One. 2014 Oct 17;9(10):e110504. doi: 10.1371/journal.pone.0110504 (PMC4201533; doi:10.1371/journal.pone.0110504)
Supplement: Table S1 — Effect of stabilized MetA protein on E.coli growth at different temperatures or in the presence of sodium acetate. Strains were grown in M9 glucose medium (pH 7.0 or 6.0) with or without sodium acetate (20 mM) in an automatic growth-measuring incubator at indicated temperatures for 24 h. The specific growth rate μ (h−1) was calculated through linear regression analysis of ln(X/X0) data with Sigma Plot software, where the initial OD600 (X0) was 0.1–0.15 at the zero time point, and X represents the OD600 values measured every 10 min in an exponentially growing culture over 1 h. (DOC) [file pone.0110504.s001.doc]

**Supporting Information to**

**Stabilization of the Homoserine-o-Succinyltransferase (MetA) Decreases the Frequency of Persisters in *Escherichia coli* under Stressful Conditions**

Elena A. Mordukhova and Jae-Gu Pan*

Superbacteria Research Center, Korea Research Institute of Bioscience and Biotechnology (KRIBB), Korea

*Corresponding author

Tel. +82428604483, [jgpan@kribb.re.kr](mailto:jgpan@kribb.re.kr)

Table S1. Effect of stabilized MetA protein on *E.coli* growth at different temperatures or in the presence of sodium acetate.

| Strain | Specific growth rate μ ( h^-1^) at | | | | | |
| --- | --- | --- | --- | --- | --- | --- |
|  | 37^0^C  pH7.0 | 42^0^C pH7.0 | 44^0^C pH7.0 | 45^0^C pH7.0 | 37^0^C,  pH6.0 | 37^0^C, pH6.0  20mM NaAc |
| WE | 0.71 | 0.76 | 0.079 | ND | 0.53 | 0.18 |
| WE-LYD | 0.73 | 0.77 | 0.53 | ND | 0.56 | 0.21 |
| WErph^+^ | 0.82 | 0.86 | 0.58 | 0.079 | ND | ND |
| WE-LYDrph^+^ | 0.82 | 0.87 | 0.65 | 0.44 | ND | ND |
| W3110 | 0.71 | 0.76 | 0.55 | ND | ND | ND |
| W3110-LYD | 0.72 | 0.79 | 0.66 | ND | ND | ND |

Strains were grown in M9 glucose medium (pH 7.0 or 6.0) with or without sodium acetate (20 mM) in an automatic growth-measuring incubator at indicated temperatures for 24 h.

The specific growth rate μ (h^-1^) was calculated through linear regression analysis of ln(X/X_0_) data with Sigma Plot software, where the initial OD_600_ (X_0_) was 0.1-0.15 at the zero time point, and X represents the OD_600_ values measured every 10 min in an exponentially growing culture over 1 h.
